# On the Causes and Treatment of Abortion and Sterility

**Published:** 1847-10-01

**Authors:** 


### ON THE CAUSES AND TREATMENT OF ABORTION AND STERILITY: being the Result of an extended practical Enquiry into the Physiological and Morbid Conditions of the Uterus, with Reference especially to Leucorrhœal Affections and the Diseases of Menstruation. By *James Whitehead*, F.R.C.S., Surgeon to the Manchester and Salford Lying-in Hospital. 8vo, pp. 426. London and Manchester, 1847.

THE simple title of this work gives a very imperfect idea of its contents. The subject of Sterility occupies a mere fraction of space, and upwards of one-half of the whole volume is taken up with an elaborate account of Menstruation as a physiological process, and of the disorders which its deviations from health are apt to produce. That there is a good deal of repetition and unnecessary prolixity in the details on many of the topics that are discussed, must be admitted. Nevertheless the work is, on the whole, valuable and instructive, and one that reflects much credit alike on the industry and practical skill of the author. It contains ten chapters, of the leading contents of which we shall now endeavour to give a faithful summary, reserving most of our comments to the close of the article.

Chap. I. is devoted to the subject of Menstruation, more especially in reference to the physical properties of the menstrual flux, and to the source or seat from whence it is derived. As the properties of this fluid, when it escapes from the body, are believed to be very essentially modified by admixture with the secretion of the passage along which it is discharged, it will be useful briefly to notice the latter in the first place.

*Nature of the Vaginal Mucus.*

"The mucus of the vagina, in its normal state, always exhibits acid properties; that of the uterus is as constantly alkaline. In no instance have I found the secretion of the vaginal mucous surface produce an alkaline reaction, except in gonorrhœal affections, and in inflammation from other causes, resulting in the secretion of pus. On the other hand, the discharges from the interior of the uterus, whether diseased or healthy, with the exception of those of an ichorous nature, have been, in every instance where I have had an opportunity of testing them, invariably alkaline. In cases of retention of a clot of blood or of a piece of the placenta, or where a portion of the ovum or the whole of it, remains for a length of time unexpelled, after it has ceased to exist, a circumstance which often happens during the early months of pregnancy, the discharge exhibits acid properties, emitting at the same time an offensive odour: this obviously arises from the process of putrefaction, by which these morbid fragments are frequently thrown off.

"Mucus like albumen has the property of coagulating on the addition of an acid, owing, probably, to a small proportion of albumen which, in its healthy state, it is always found to contain. Mucus, however, as well as albumen, wil bear the admixture of a small quantity of acid, or of alkali, without undergoing any change in its appearance. But the acid secreted along with the vaginal mucus is, under normal circumstances, in quantity sufficient slightly to coagulate it, and in this state the fluid is generally found; except during menstruation, or when the uterine mucus happens, which is extremely rare, to be produced in unusual abundance.

"The parts being in a perfectly healthy condition, the whole surface of the vaginal membrane is covered by this form of mucus, which, when in very small quantity, is transparent; but in places where it is found collected in larger quantity, and especially when collected within the speculum tube during its introduction, it has a milky appearance, is perfectly opaque, and much less viscid than other mucus. It is the mixture of this acid product with the true catamenial fluid in its transit outwards, which gives to the latter the peculiar properties by which it is generally said to be characterized." P. 20.

The acid in the vaginal mucus is, as we shall presently see, said to be similar in its properties to the Acetic. It "enters readily into combination with the protein compounds contained in the blood and the secretions, dissolving them in all proportions, and communicating to the product with which it is associated, strong antiseptic properties. Moreover, it deprives the vaginal mucus of the viscidity which other mucus possesses, and thus renders it less liable to thicken and accumulate between the folds of the membrane; and it is to this agency, likewise, that effused blood may be for a much longer period arrested in the vagina than within the uterus, without undergoing the putrefactive change." This remark will prepare the reader for our author's views respecting the catamenial discharge, the properties and characters of which, according to him, depend in a great measure upon its admixture with the acid mucus of the vagina. Let us first see what is the *nature of the true* (i. e. unmixed with this mucus) *menstrual secretion.* And, first, how is it to be obtained?

"For the purposes of examination, it may be procured by means of the speculum, care being taken always to remove, by the aid of a piece of lint or sponge held between the blades of a sponge-holder, all the secretion from about the *os* and *cervix uteri*. This must be done immediately after the instrument shall have been properly adjusted, and will be found necessary in order that the fluid may be free from admixture with that exuding from the surrounding surfaces. In consequence of the irksomeness of the procedure, however, the patient will seldom be able to remain in the required posture longer than until from ten to twenty grains have accumulated. This quantity, although too small for the purposes of chemical analysis, will nevertheless be sufficiently large for determining its sensible properties, as well as for microscopic investigation." P. 21.

Now, without saying anything, for the present, as to the decency of the proceeding here described, we shall only remark, that Mr. Whitehead believes, from his experiments, that the *true* menstrual secretion very nearly approximates to, if it be not identical with, the blood circulating in the capillary vessels, and that "the properties which are said to characterize *common* menstrual blood—namely, its want of power to clot, attributed to the absence of fibrin, its acid reaction, and its peculiar colour and consistence—are altogether acquired, and variously modified, by admixture with the vaginal mucus after the fluid has escaped from the uterine vessels;" the *true* menstrual fluid readily coagulating, and invariably exhibiting an alkaline reaction, as ordinary blood is found to do. It is obvious, therefore, if this view of the case be correct, that the menstrual discharge is to be regarded rather as a simple exudation than as a proper secretion.

That the change of the *true* into the *common* or *crude* menstrual fluid is owing to the action of the acid mucus of the vagina is attempted to be proved by some experiments:

"If blood that has been drawn from the basilic or some other vein, or collected from a scarified surface, be allowed to flow into a cup containing pure or diluted acetic acid,—the same acid which exists in a free state in the vaginal mucus,—in the proportion of one part of pure acid to sixty parts of blood, and if the acid be properly diffused through the blood; the latter will remain uncoagulated, and become of a darker colour if the blood be arterial, but lighter if venous, and no traces of uncombined fibrin can then be found in it." P. 25.

That the menstrual discharge proceeds from the internal surface of the uterus has been taken for granted in the preceding observations, and indeed is a *fact* that is now generally recognised in physiological science. "I have witnessed," says Mr. Whitehead, "in numerous instances the menstrual blood issuing from the *os uteri;* the labia, cervix, and all the vaginal surface being perfectly normal, and furnishing no such product. Some of these observations were made upon females affected with *procidentia uteri;* but, in the majority, the information was obtained by means of the speculum." He had an opportunity, about two years ago, of confirming these observations by a *post-mortem* examination. A young girl died from the effects of a menorrhagic attack: the hæmorrhage had ceased only 24 hours before death.

"An early post-mortem examination discovered no organic lesion in any part of the body, which was everywhere drained of blood. The uterus was rather larger than natural; its parietes were less firm, but nearly of the usual thickness. Its interior contained a clot of blood which occupied the entire cavity. This clot, which was an exact mould of the uterine cavity, measured, from its lower extremity which terminated at the *os tincæ*, to the part situated at the *fundus uteri*, two inches and a quarter; and between its two horns, one inch and three quarters. Each horn had a rounded extremity, was semi-transparent and fibrinous, and terminated at the corresponding Fallopian orifice. The Fallopian tubes were perfectly empty, and of the natural dimensions. The right ovarium was a little larger than the left, and presented several distinct cicatrices in different stages of reparation. One of these was recent, depressed, puckered, and appeared at first sight to have an opening in its centre; but it was afterwards found to be impervious. The cavity over which this was situated was occupied by a firm, reddish coagulum, traversed by yellow striæ, having an irregularly concentrical arrangement. Immediately beneath the next most distinct cicatrix was a firm yellow body, smaller in size than the preceding, and less striated. Another paler formation of a similar kind, but still smaller and more deeply seated, and marked by a faint indentation of the surface above it, was observed in the same ovary. There were also two vesicles of different sizes containing a thinnish glairy fluid, one of which was near the surface.

"The left ovarium presented on one of its sides a large bluish vesicle, projecting its peritoneal covering considerably above the surrounding surface; this was the size of a small hazel nut, and contained apparently nothing but serum. Two smaller vesicles more deeply seated, and two well-marked yellow bodies of different dimensions having corresponding cicatrices, were also observed in the same ovary.

"The *labia* and *cervix uteri* were perfectly healthy. The inner surface of the uterus presented numerous openings scattered over every part of it, obvious to the naked view, some being sufficiently large to admit a good-sized bristle, or the end of a lachrymal probe. The largest and most numerous were at each side of the fundus near the horns of the uterus, and at the contracted part of its body near the commencement of the cervix. The openings had a valvular arrangement, a great number passing downwards towards the cervix, while those at the upper part of the organ appeared to pass towards the Fallopian orifices." P. 35.

The appearances, discovered in this case, are fairly regarded by our author as confirmatory of the opinion that menstruation is in short nothing more than "a simple exsudation of blood from the arterial capillaries in communication with the valvular orifices naturally existing upon the inner surface of the uterus."

But, if the *true* or proper catamenia invariably proceed from the inner surface of the womb, it is to be borne in mind that a sanguineous discharge, having the very same appearance, and possessed of the same physical properties, and returning, moreover, at regular monthly intervals, may proceed altogether and exclusively from the labia and outer portion of the cervix of the uterus, and this too whether the cavity of that organ be occupied with the product of conception or not. It is to this state of things that our author applies the term of *spurious menstruation*. And now comes the important (most important, certainly, if confirmed by subsequent enquiries) ætiological position, which he labours to maintain; viz. that "it (the discharge in question) is invariably associated with a morbid condition of the parts external to the cavity of the uterus, generally of its cervix and labia; sometimes of a portion of the vaginal mucous surface." Several facts are adduced in the way of proof: we shall presently have occasion to allude more particularly to them.

It has been already stated, that pregnancy may co-exist with this spurious form of menstruation. Not unfrequently, however, it is a mere sham or mock pregnancy that is present under such circumstances. This state is thus described by Mr. Whitehead:—

"Spurious menstruation occurring in the absence of pregnancy is accompanied with enlargement of the abdomen and of the mammary glands, nausea and occasional vomiting, alternate rigors and flushes of heat, languor, loss of rest, precarious appetite, and other symptoms tending to encourage a suspicion of the existence of pregnancy. The abdomen is sometimes enormously and painfully distended, as if caused by accumulation of flatus, and then suddenly subsides, but seldom to the dimensions of the unimpregnated state; there is constant aching of the loins, hips, and hypogastrium; a sense of bearing-down, and inability to retain the urine the ordinary length of time. Pressure of the distended parts upon the large arterial trunks produces a sensible throbbing over the whole region, which, together with the visceral movements consequent upon the shifting of the confined flatus from one portion to another of the lower bowel, strengthens the belief in the existence of a fœtus in utero; and it is often a very difficult matter to convince the patient, under such circumstances, that she is not with child." P. 37.

Three examples are related in illustration of these remarks; but we must candidly confess that, while we admit that they certainly attest the importance of examining the state of the uterus with the speculum in some obstinate and doubtful cases, we are by no means quite satisfied that the monthly-recurring sanguineous discharge in all of them was not the genuine catamenia, and merely an exsudation of blood from the ulcerated surface of the cervix uteri; as is alleged by our author, in consequence of his finding upon ocular inspection that the *os uteri* appeared to be closed and linear. That the reader however may judge for himself, we shall give the report of one case entire, especially as its details will serve to illustrate some other points, which will subsequently come under our noticc.

"Sarah Neale, a married woman, twenty-six years of age, was admitted a patient of the Manchester Lying-in Hospital in January, 1846. She had borne two living children at the full term of gestation, and subsequently, one still-born at the end of the seventh month. At the time of her admission she stated herself to be in the seventh month of her fourth pregnancy; and sought relief at this period for an abundant leucorrhœal discharge which had existed ever since her miscarriage, and which she believed to be the result of a gonorrhœal affection, contracted from her husband, when between five and six months advanced in that pregnancy. To this cause she referred her premature delivery. Although nearly seven months advanced, she had felt the fœtal movements only very slightly and at long intervals; and she had menstruated regularly every month during the whole period, the discharge being precisely the same both as to its appearance, the number of days during which it continued, and the attendant symptoms, as when not pregnant. She complained of unusual weight and fulness of the abdomen, a fixed pain of the right hypogastrium, constant aching of the loins, and a sense of bearing down. The vaginal discharge which, in the absence of menstruation, was a yellowish matter, communicating a deep stain to the linen, exhibited a faint acid reaction, but was sometimes decidedly alkaline. The abdomen was of medium size, somewhat larger on the right than on the left side, and the skin appeared a good deal loaded with adipose deposit. The mammary glands were enlarged, and the areolæ and follicles more than ordinarily distinct. A tumour, which appeared to be the uterus, was sufficiently palpable above the pubis; but no fœtal impulse was communicated, on careful manipulation, to the hand; nor could the placental *soufle* or the beat of the fœtal heart be detected. To the touch, the uterus, although somewhat enlarged, was loose and floating; its cervix hard and hypertrophied, and the *labia* expanded and irregular. Examined with the speculum, the *os uteri* was found perfectly linear, and closed; the whole circumference of the *labia* was one mass of granulations of a purplish colour, covered with pus, and exuding a little blood from several points, caused probably by temporary pressure of the instrument. The adjacent vaginal reflection was thickened and varicose. My opinion was at once given that pregnancy did not exist, which, however, the patient was unwilling to believe.

"During the following menstrual crisis I examined the parts again with the speculum. The ulcerated surface was covered with blood, which, being carefully removed by means of lint, the parts were brought more distinctly to view. The *os uteri* was still completely closed, and gave no escape to any fluid; but the diseased parts were again covered with a sanguinolent exudation, and this was repeated as often as the product was cleared away; the uterine orifice still remaining closed and free from discharge as before. A course of alterative medicine, with repeated small bleedings by leeches from the hypogastrium, and, subsequently, the application of nitrate of silver to the ulcerated surfaces, effected a complete cure. The abdomen gradually subsided, the pain and bearing-down disappeared, and the menstrual function was restored to its normal state." P. 39.

It will be observed that the " normal state" here mentioned alludes not to any alteration in the physical properties, or in the periodic recurrence of the discharge, but simply and altogether to its seat or origin, as discovered by the use of the speculum.

CHAP. II.—*Conditions which principally influence Menstruation at its commencement.*

The following table is given by Mr. Whitehead, to shew the age at which this function was established in 4000 individuals.

| | | | | |
|---|---|---|---|---|
| " At the age of | 10 | years, | 9 | first menstruated. |
| " | 11 | " | 26 | " |
| " | 12 | " | 136 | " |
| " | 13 | " | 332 | " |
| " | 14 | " | 638 | " |
| " | 15 | " | 761 | " |
| " | 16 | " | 967 | " |
| " | 17 | " | 499 | " |
| " | 18 | " | 393 | " |
| " | 19 | " | 148 | " |
| " | 20 | " | 71 | " |
| " | 21 | " | 9 | " |
| " | 22 | " | 6 | " |
| " | 23 | " | 2 | " |
| " | 24 | " | 1 | " |
| " | 25 | " | 1 | " |
| " | 26 | " | 1 | " |
| | | Total | 4000 | |

" Average age of the first menstrual crisis, fifteen years and nearly seven months." P. 46.

In treating of the influence of the habit or diathesis of the system on menstruation, a good deal of space is most unnecessarily occupied with common-place remarks on the general physiological subject of temperaments; and, not satisfied with this discussion, our author actually proceeds to discuss the origin and ætiology of Scrofula. That his reasoning is occasionally apt to be rather loose and illogical on some of the questions he undertakes to solve is pretty apparent from the story, which is recorded

"to show in what manner Scrofula may be induced by the action of mercury, independently of previous disease."

Mr. Whitehead is certainly not warranted, according to general experience, in attributing the development of scrofulous disease so frequently as he seems inclined to do to a venereal taint of the system. No sooner is this subject broached than forthwith he proceeds to narrate cases of syphilitic disease in infants being followed, at a subsequent period, by the appearance of scrofulous cachexy. If space permitted, it would be easy to shew the inconsequential nature of several of the assertions. He is probably more correct, when he impugns the accuracy of many of the statements, that have been published by Commissioners and others, touching the injurious effects of factory labour upon the health of young females.

"It has been asserted for instance—and the assertion is quoted in every European country as referring to an absolute fact—that factory employment has a particular tendency to induce premature development of the sexual organs, and that a precocious womanhood is more frequently witnessed among the manufacturing population than among other classes of people. Until lately, I also entertained a similar belief, based, it must be confessed, upon the authority of others whose statements I now believe to have been advanced at random, or founded, at best, upon a very few isolated cases, altogether inadequate to a general conclusion. A more extended inquiry into the subject has convinced me of this popular fallacy. The subjoined facts will show that, in this respect, mill employment has an effect the very opposite of that which it is generally believed to have.

"Of the 4000 individuals before quoted, 2127 were employed in mills, warehouses, and places connected with the various processes of manufacturing, at the period of puberty and for a length of time previous to its advent. Their collective ages amounted to 33,296, affording an average of *fifteen years*, and nearly *eight months*; of whom 511, or 24·02 per cent. suffered under disease consequent upon undue retardation of the functional changes at the commencement.

"The remainder, 1873 in number, included all that were otherwise circumstanced; as domestic and farm servants, hawkers, sempstresses, and shopwomen; together with a number of educated females not necessarily engaged in any pursuit, except school exercises. The sum of their ages was 28,982, giving *fifteen years* and about *five months* as the average age of puberty; and 375, or 20·02 per cent. experienced functional difficulty in form of amenorrhœa at the onset." P. 81.

Mr. Whitehead declares, as the result of extensive observation, that the high temperature maintained in mills is far less injurious to health than has been represented by many writers. Nay, he does not hesitate to affirm that, were it not for the hurtful effects of exposure to a cooler, and often a cold, temperature upon leaving the mills, "he feels persuaded that a more efficient or more valuable auxiliary could scarcely be employed in these latitudes, in the treatment of some strumous affections than this artificial climate is capable of affording."

According to our author's observations, young females, the daughters of persons in easy circumstances, and who are therefore not engaged in any other occupation save the usual educational exercises, arrive at the epoch of puberty, as indicated by the advent of the catamenia, somewhat earlier than those who are labouring at some manual work. In the former, the change took place at about the age of 14 years and a half, "being more than a year earlier than the general average; and they suffered under disease consequent upon functional difficulty at the rate of 32·30 per cent., a proportion far exceeding that of any of the classes already noticed."

Every medical man will recognise the soundness of the following remarks:

"The cause of this peculiar susceptibility to disease in the class of patients now under consideration, becomes a question of paramount importance; and is, without doubt, to be found in the faulty system of education pursued at boarding schools. The mental faculties are called into premature activity, and tasked to a degree beyond their power of endurance, and the mind is crowded with a mass of matter under the fictitious denomination of accomplishments, which Nature never designed that it should entertain; while the proper training of the physical constitution is totally disregarded. Moreover, the indulgence of a propensity, irresistably active at this period of life, to the reading of novels, romances, and works of a similarly vicious character, has likewise a very injurious tendency. Witness for instance, the violent and often dangerous paroxysms of hysteric emotions occasionally consequent upon the mere perusal of an affecting narrative, having probably no foundation save in overstrained analogy, or in fiction. These are agencies to which the unlettered are entire strangers, or which they experience but seldom, and only to a very limited degree.

"It thus appears that an occupation of whatever kind, and wherever pursued, which requires no more than a moderate application of mere physical power for its performance, will bear a very favourable comparison in regard to its effect upon bodily health, with one in which the exercise of these faculties is neglected. The fact is, the muscles were designed for a purpose; and their due employment is no less necessary to the harmonious working of the economy of which they constitute an important portion, than for maintaining a healthy relationship with the objects of external nature. An employment requiring moderate and regular exercise, especially if pursued in an open untainted atmosphere, is of the first importance; while inactivity, whether the individual reside in town or country, is almost invariably followed by ill consequences. In no class of people is the menstrual function so healthily performed as in those necessitated to earn the means of subsistence by personal exertion; provided always that the employment be suitably adapted to the constitutional powers of the individual; while the converse of this is known to obtain with those in easy circumstances. Amongst farm servants and others, the period of puberty is seldom accompanied by any derangement of the general health; but in the higher grades of society, it forms an era of the greatest anxiety, and is often the commencement of serious and irreparable organic mischief." P. 90.

### CHAP. III.—*Diseases of Menstruation.*

There is much in this chapter too, that might have been omitted without any detriment. A great portion of the details, connected with the subject of Amenorrhœa, serves only to swell the bulk of the volume. Witness, for example, the minute details of six or seven cases recorded to illustrate the effects of retarded mentruation in young girls, and of others to shew the ill consequences of the sudden stoppage of the discharge from exposure to wet and cold, or from some violent emotion of the mind, &c.

The account given of Dysmenorrhœa is exceedingly incomplete. Mr. W. alludes to the occasional presence of ulceration of the *os uteri* in this morbid state, but without attributing the one to the other as its producing cause. Dr. H. Bennett, however, has recently endeavoured to shew that, even in virgins, painful menstruation is occasionally connected with structural lesion of the uterine orifice, that a correct diagnosis cannot be formed without the aid of the speculum, and moreover, that the disease cannot be effectually cured without appropriate topical treatment. He does not hesitate even to propose that the hymen should be divided, when the speculum cannot be easily introduced. Surely such an extreme measure as this is scarcely warrantable under almost any circumstances; but, as Dr. B. has promised to publish the particulars of some cases in which he deemed its adoption necessary, we shall only, for the present, express our earnest hope that no medical man will ever have recourse to the step in question, save with the sanction and concurrence of another professional man, as experienced at least as himself.

We are uncertain whether Mr. Whitehead has used the speculum in virtuous unmarried females. No distinct mention is made of it in the body of his work; but there is a strange allusion to it in the introductory remarks, in these terms:—"Some are admitted (into the Manchester Lying-in Hospital) who are neither pregnant nor suckling, as well as a number of young unmarried females, labouring under retention or suppression of the menses, leucorrhœa, prolapsus, hysteria, chlorosis, and similar affections connected with disordered menstruation. In all such cases, I made it a point, whenever practicable, to examine the uterus with the speculum, and almost invariably found the existence of disease of this organ sufficient in extent to bring the other symptoms under the arrangement of sympathetic disturbances merely. This view was confirmed by the results of the treatment adopted. I have now examined upwards of two thousand such cases, and the result has almost invariably been the same." Are we to understand from this passage that there is organic or structural disease of the uterus in all cases of "retention or suppression of the menses, leucorrhœa, hysteria"? &c. Surely not; the very supposition would be monstrous.

The section on *Menorrhagia*, or, as our author calls it, Metrorrhagic Menstruation, contains but little that calls for notice. Considerable confusion has been most gratuitously introduced into our author's remarks by his using the term *metrorrhagia* in a different sense from metrorrhagic menstruation; the former he employs to designate "uterine hæmorrhage, altogether unconnected with menstruation." It must be to this description of sanguineous evacuation that the following remarks on "metrorrhagic discharges" apply:—

"These are not unfrequently witnessed in young girls before the age of puberty; and they are by no means uncommon in those who have finally ceased to menstruate. In the former, such phenomena are usually associated with the hæmorrhagic diathesis; in the latter, with disease of the uterus, which, when not of a malignant character, consists in ulceration about the *cervix*, often the result of varicose inflammation; or of *endo-uteritis*." P. 141.

Whether the "abnormal discharges of blood," referred to in a subsequent paragraph, belong to metrorrhagia exclusively, or whether they apply in part to metrorrhagic menstruation, it is not quite easy to determine:

"Abnormal discharges of blood frequently come on during the early months of pregnancy; or supervene upon suspension or irregularity of the menstrual function, arising from other causes, and being accompanied with abdominal enlargement and the general indications of pregnancy. When this state of things happens in the young unmarried female, the investigation is often fraught with peculiar difficulty: the employment of the only means by which a satisfactory knowledge of the case can be arrived at, being obstinately objected to. And even should every facility for the procedure be afforded, whether the existence of pregnancy be substantiated or disproved; it is still one of the most delicate positions in which either patient or practitioner can be placed, involving, on the one part, the discharge of a very disagreeable, difficult, and often thankless duty; on the other, sacrifice, to some extent, of character, and perhaps exclusion from society. For although the calumny which led to the investigation be proved to be unfounded, it is difficult to remove altogether the impression from the mind of the multitude: a woman's virtue cannot bear even to be suspected. Should an unfavourable opinion be pronounced and be found erroneous—for after the most rigid scrutiny, the ablest enquirer may fail to discover the truth—the mistake will never be forgotten, but will remain a blot upon his character ever afterwards. Under all circumstances, the practitioner cannot be too scrupulously guarded in delivering an opinion; and it should ever be his aim, however culpable his patient may be, to shield her fame, as far as is practicable, from the censorious taunts of an unfeeling public." P. 144.

Mr. Whitehead has omitted to inform his readers how this awkward blunder may be best avoided.

### Chap. IV.—*Last Menstrual Crisis.*

The particulars are given of a case, in which menstruation continued to be regularly and uninterruptedly performed from its first establishment in early life up to the 75th year. The woman was, at the beginning of the present year, an inmate of the Manchester Union Hospital. "It is remarkable that during every pregnancy (13 in all) she continued to menstruate until the last month of the full time, the discharge being similar in quantity and character to that of the non-gravid state; and that the discharge re-appeared, notwithstanding that she suckled her children, in two or three months after the birth of the child."

"With the exception of the pregnant periods just referred to, the menstrual discharge has suffered no interruption from its commencement until the present time, and there have been no particulars in which it has differed during the latter years of life from its state during her youth. At both times it continued for three days; and now, as then, it is preceded and accompanied by a sense of lassitude and pain in the back, which apprise her of its approach. The character, too, of the discharge as it occurs now, does not differ either in appearance or quantity from that to which she has been accustomed throughout life. She further states that her mother, who attained to a great age, was the subject of the same peculiarity; continuing to menstruate until the close of life; and that she had always heard a similar account of her maternal grandmother."

Mr. Whitehead mentions a still more curious case, in which a regular monthly discharge occurred in an old lady, 91 years of age, for upwards of a twelvemonth before her decease, although she had ceased to menstruate in her 54th year.

"In February, 1845, thirteen months before her decease, having suffered for several weeks previously from distension and general uneasiness of the abdomen, aching of the loins, and slight dyspeptic symptoms, a quantity of blood, bearing all the characters of ordinary menstrual fluid, was observed escaping from the vagina, and at first occasioned considerable alarm. It came on in the night-time, an hour or two after she had taken, with the view of promoting perspiration, a cup of hot beer-whey, and it continued uninterruptedly three or four days, then ceased; leaving the patient perfectly free, however, from the pain and uneasiness before complained of. About three weeks afterwards a similar train of symptoms appeared; the discharge, preceded by the lumbar and abdominal uneasiness as on the former occasion, continuing from three to four days, after which she expressed herself completely restored. In this manner the functional phenomena were periodically developed at intervals of twenty-three or twenty-four days, during the remaider of her life; the health being in a good state the whole time, with the exception of some slight sympathetic disturbance at the critical periods. She died at the thirteenth or fourteenth accession, while the organic product was still escaping. On each occasion the discharge, both as to quantity, appearance, and the period of its duration, together with the premonitory and accompanying symptoms, were everyway similar, so far as she was able to judge, to what they had formerly been during the activity of the catamenial function. An inspection of the body, after death, was not permitted." P. 148.

In another case, related by our author, menstruation has continued to recur regularly up to the 66th year; the lady is still alive.

From a tabular list of 69 cases, of whose history a brief abstract is given, it appears that the average age, at which menstruation ceases in this country, is between the 47th and 48th years of life. With respect to the diseases which more immediately appertain to this important change, our author remarks:—

"The forms of uterine disease commonly met with at the period of the last change of life, may, for practical convenience, be arranged under three heads, each distinguishable from the other by the character of the discharge with which, early or late, it is almost invariably accompanied; and also, by the kind and degree of suffering which it occasions in the organ immediately implicated, and the sympathetic disturbances simultaneously awakened in remote parts of the body. The *first*, and most common of these affections, is characterized by a muco-purulent discharge from the vagina, generally denominated *leucorrhœa*, or the whites; but which differs from simple leucorrhœa in several important particulars; the *second*, is often accompanied by vaginal hæmorrhage, the discharge differing, both as to its properties and in its source, from the menstrual product; the *third* form, is characterised by a watery, sanious, serous, or ichorous discharge, which is sometimes mixed with blood, sometimes with pus, mucus, or albumen-like shreds, and occasionally with small portions of fleshy matter, the product of the organic change upon which it depends. It generally emits an offensive odour." P. 155.

The *first* of these states is almost always associated with, and indeed dependent upon, the morbid state of the womb that is thus described:

"The lesion usually consists in hypertrophy of the *cervix*, or of the whole organ, with an ulcerated state of one or both *labia*. The most common form of ulceration is that of a simple granulating surface with a defined margin. Sometimes there is induration of the anterior *labium*, which, when considerably enlarged, projects downwards in a conical form, and is generally excoriated or superficially ulcerated, especially on its posterior surface; the abrasion extending in an upward direction towards the inner *cervix*. At other times both labia are indurated, with or without abrasion; and, accompanying this state of parts, there is often also an ulcerated fissure, more or less deep, occupying one or both *com*-

*missuræ labiorum.* Occasionally, the *labia* appear but little, or even not at all enlarged, or in any way diseased, but the margin of the *os* presents a ring of intense redness. This is the sure indication of inflammation of the lining membrane of the uterus, or *endo-uteritis*; and is accompanied with enlargement of, and pain upon pressure being applied to, the body of the organ. That these morbid conditions of the uterus constitute the principal cause of all the disturbances before enumerated, is sufficiently clear, from the circumstance of their being most signally, often instantaneously relieved, by the mere use of local remedies, even without the assistance of constitutional treatment." P. 158.

For the cure of such cases, the general health must be improved by the use of alteratives and tonics ; the local disease will require the occasional application of the nitrate of silver, either in the solid state or in that of a very strong solution—ʒj. to ʒiij. of water.

When there is reason to believe that chronic Endo-uteritis is present, the treatment recommended by our author is thus explained :

"It consists in the application of leeches to the *hypogastrium* or over the upper part of the *sacrum ;* and in the exhibition of remedies similar to those employed in Case XXI. In addition, when all febrile excitement has become completely subdued, a more decidedly tonic plan will probably be indicated ; and considerable benefit may be derived from injecting within the *uterus*, a weak solution of nitrate of silver in combination with *extractum conii* ; or by the introduction of an ointment of the same material. The latter form of the remedy is applied by means of a small piece of lint fixed upon the end of a long probe, and fastened to the handle of the instrument with a thread, with a view of securing its safe withdrawal. Not only does no disturbance or discomfort of any kind ensue upon the employment of these local measures ; but, on the contrary, a beneficial change is often evident after the first application, and I have witnessed the suspension of pain to be instantaneous. The operation of injecting is done with the aid of the speculum, by means of a long syringe similar to Clarke's female syringe, having a nearly straight tube, with but one hole at its extremity. If carefully managed, from one to two drachms of the remedy, (which may be composed of different materials, varied in form and strength according to circumstances)—will pass within the organ at each operation, most of which returns almost immediately. The temperature of the fluid, previous to being used, should be raised to about ninety degrees, Fahr." P. 163.

The *second* condition alluded to is, in very many cases, connected with the existence of a varicose ulcer of the cervix of the uterus. "The hæmorrhage, which is sometimes alarmingly profuse, may cease after a few hours ; but it often continues many days, or weeks, and sometimes several months. On subsiding, it is replaced by a light-coloured discharge which is observed, occasionally, to be more or less mixed with blood, but consisting essentially of a combination of mucus and pus in variable proportions ; the vaginal mucous membrane participating, in greater or less degree, in the generally increased action of the surrounding parts, thus furnishing its peculiar secretion in unusual abundance. The affection under this form, familiarly known as 'the whites,' may harass the patient for years, or, by gradually undermining the constitutional powers, may keep her in a state of suffering and misery to the end of life. In some instances the congestion, which at the onset was confined to the lower part of the organ, does not terminate in varicose ulceration, but, becoming more generally diffused, assumes the form of uterine phlebitis, accompanied with abdominal enlargement, and alarming constitutional disturbance. Inflammation of the peritoneum is often a consequence of the further extension of this state of the affection, with effusion into the peritoneal cavity, constituting a common form of abdominal dropsy."

As an illustration of this varicose ulcer of the uterus, we may take the following instance:

A poor woman, 50 years of age, and who had been 14 times pregnant, ceased to menstruate in her 48th year. Five months afterwards, "the patient being in a very infirm health, with abdominal enlargement, creating a belief in the existence of dropsy, a profuse uterine hæmorrhage came on, which passed away in clots of small size. This continued in a moderated degree, without ceasing, more than four months, when it was replaced by the leucorrhœal discharge, in its accustomed form. Her linen, which she exhibited, was covered with thick, yellow stains, and the recent secretion was strongly alkalescent. She complained of a fixed, deep-seated pain of the right hypogastrium, near the groin; severe and constant aching of the right hip and thigh, and across the sacrum; frequent desire to void the urine, with sense of constriction in the region of the bladder; disordered digestion, and irregular bowels; attacks of alternate chills and flushes of heat; and great physical prostration. Examined with the speculum, the *cervix uteri* was found unusually tumid, and was traversed by a number of prominent venous branches of different sizes; the anterior *labium* was hard, hypertrophied, and excoriated; the posterior *labium* was also enlarged, and completely occupied by a flabby, purple-looking sore with irregular surface, from which a small quantity of blood oozed out during the operation. The treatment consisted in the application of leeches to the hypogastrium; and subsequently in the use of the solid nitrate of silver to the ulcer and to the whole of the lower part of the uterus. She also took, night and morning, a grain of calomel combined with half-a-grain of opium, until the mouth became slightly affected; and a dose of the compound decoction of sarsaparilla, containing three grains of iodide of potassium, thrice daily for six weeks. The nitrate of silver application was four or five times repeated, at intervals of a week; and after relinquishing the sarsaparilla, she was ordered to take small doses of quinine and aloes, for several weeks more. The improvement progressed favourably from the first. She was discharged cured, on the 15th of July, three months after her admission; but appeared sufficiently recovered several weeks earlier."

Of the *third* morbid state to which we have referred, it need scarcely be remarked that fætid sanious discharges from the uterus, occurring in women advanced in life, are justly considered as generally indicative of some serious organic mischief of the uterus. No prudent medical man, however, will even venture to give a definite opinion upon such a case, until he has been permitted to have not only a digital, but a specular, examination of the suspected parts. "Diseases of a malignant character," says our author, "are, generally speaking, preceded and attended by a similar train of symptoms, both constitutional and local, in each case; varying of course from time to time, according to the nature of the structure implicated, and the extent and severity of the local affection. A notable preliminary condition is a plethoric state of the portal system of vessels, with occasional functional impediment of the organs in immediate relation therewith, and especially with symptoms denoting determination towards the region of the uterus. The final catamenial change is remarkable for profuse menstrual and leucorrhœal discharges, the latter usually persisting after the former has ceased to appear. Hæmorrhoids are a common affection under this state of the system ; disordered digestion ; pain of the head, especially at its summit and back part ; erysipelas of the face ; anasarca ; disordered state of the urinary organs ; and sometimes partial paralysis."

In the narration of a case of the "cauliflower disease" of the cervix uteri, it is remarked that "the speculum, the employment of which was so strenuously objected to at the onset, became the medium of administering, in the most gratifying manner, to the patient's comfort during the latter months of her life. The sore, by its aid, being fully brought within view, the whole of the accumulated, offensive secretion could be effectually removed, and the surface treated with a soothing anti-septic agent, which rendered her comparatively comfortable for a length of time afterwards. These applications, which were composed of chloride of lime combined with *opium*, sometimes with extract of *conium* or of *belladonna*, became necessary every second or third day. On two or three occasions, when the operation was too long delayed, she experienced an attack of shivering, with increased pain of the *sacrum* and *hypogastrium*, vomiting, headache, and hectic fever. Such accessions were frequently repeated during the last few weeks of her life, and were always found to be most severe when a portion of the ulcerated surface had beeome changed (which was several times observed,) into a black, powdery, melanotic matter of a putrid and extremely offensive odour. This deposition could always be completely removed by means of lint held between the blades of a sponge-holder ; and it was, doubtless, owing to the timely removal of this putrid substance, and the application of powerful anti-septic remedies, that life was prolonged and rendered less painful : the melanotic change being thus prevented from implicating the deeper structures of the organ."

In the Chapter (VI), on the Signs of Pregnancy, two cases are related where, in consequence of co-existent ascites, the presence of pregnancy was not discovered even up to the last moment ; and, in one, the patient was tapped only a few hours before "a full-grown living child was expelled." As the patient, in whom this unhappy accident occurred, recovered favourably, we may very fairly dissent from our author's opinion, that there could be "no doubt that the uterus was pierced by the instrument used in the operation, and that the fluid drawn off was the *liquor amnii*, as no evacuation of the kind by the natural passages preceded or accompanied the expulsion of the fœtus."

That the inexperienced may not be too much cast down by an error of diagnosis in such a complication, it may be some satisfaction for them to learn that, in the preceding case, no fewer than three medical men "of high standing in the profession" were most grievously mistaken.

Passing over our author's remarks as to the probable cause of Quickening, we proceed to notice what he says respecting the

*Appearance of the Os Uteri during Pregnancy.*

"The only test capable of revealing with certainty the existence of pregnancy during its early stages—from a few days after conception to the middle or end of the fourth month, when auscultation first becomes available—is that which the appearance of the os uteri presents to specular examination. It was before stated that, during menstruation, the labia uteri were in a state of high vascular turgescence, and the os tincæ, although elongated, and having its boundaries somewhat relaxed, was nevertheless closed and linear, except during the escape of the small menstrual clots before noticed. At the time of conception, the parts are thrown into a precisely similar condition; but no escape of fluid occurs to relieve the turgescence, which consequently continues to increase. In from ten to twenty days afterwards, the whole organ is found considerably enlarged, and the circulation through it augmented both in force and volume; the labia are thickened and apparently elongated, the commissures less distinct, and the *os* appears to be sunk in, or dimpled, owing to the distension and consequent projection of the labia below the level of the orifice. In the fourth week, the *labia* at the centre of their margins, are permanently separated to the extent of one or two lines; and the *os tincæ,* which was before a mere chink with parallel boundaries, is now seen to be an elliptical, or sometimes rounded aperture, which is occupied by a deposition of transparent, gelatinous mucus. At six or eight weeks it becomes decidedly oval, or irregularly circular, with a puckered or indented boundary, having a relaxed and lobulated character." P. 202.

"These changes of form of the lower uterine orifice are evidently owing to distension of the surrounding textures, caused by the increased flow of blood into their structure. The whole circumference of the cervix is enlarged in all its dimensions; the labia become less and less distinct by the simultaneous expansion of the commissures, so that at the stage above-mentioned the existence of the latter is altogether obliterated. After this period, the parts present a great variety of appearances, depending principally upon the state of the circulation through the uterine veins. The characteristic trait, however, is always maintained: namely, the patulent state of the orifice, occupied by a transparent gelatinous plug of mucus, and its relaxed, irregular boundary." P. 203.

That Mr. Whitehead is quite right in attaching so much importance to the alterations in the uterine orifice, here described as one of the surest signs of pregnancy during the first ten or twelve weeks of gestation, is more than probable. Some of the best obstetrical authorities of the day agree with him. Dr. Rigby, for example, in his excellent system of midwifery, says that "he is inclined to think that the soft feel of the portio vaginalis (of the os uteri) is one of the earliest signs of pregnancy which can be detected by examination:" the transverse fissure or slit of the orifice being, at the same time, rounder and more depressed than it is in the unimpregnated state. And, in another passage, he expresses himself still more emphatically. "In a primipara, the changes, which pregnancy produces upon the os and cervix uteri, are generally sufficient to lead to an accurate conclusion. The round dimple-like depression which the os uteri forms, the soft cushiony state of the cervix, are changes which we consider as peculiarly the effects of pregnancy; but their distinctness and certainty cease when the patient has had several children; the irregular shape of the os uteri—its thickened edges, hard here and there—the os tincæ itself more or less open—the cervix scarcely, if at all, shortened, even at a late period of gestation, tend not a little to perplex the diagnosis furnished by this mode of examination; and where disease is complicated with pregnancy, the difficulty is greatly increased, and not unfrequently so much, that scarcely a single satisfactory point will be obtained."

It may be here worthy of remark, that the purple colour of the mucous lining of the vagina at its external orifice, which has been so much dwelt upon by some continental obstetricians as a sign of pregnancy, has attracted but little attention in this country; chiefly, we believe, from the unwillingness of our medical men having ever recourse to indecent exposure, when not absolutely necessary.

After Quickening has taken place, changes in the form and dimensions of the *os uteri* are not sufficiently characteristic to enable the physician to determine the exact period of the existing pregnancy; "the orifice is sometimes as widely dilated, and its boundaries as much relaxed at the end of the fourth month, as it usually is two or three months later."

Examination with the finger alone can very seldom be trusted to, to determine the existence or non-existence of pregnancy from the state of the uterine orifice; and for these reasons:

"The boundaries of the *orificium uteri* are not constituted of the identical material which formed the margin of the *os tincæ* of the unimpregnated uterus. They consist of the loose cellular and superjacent mucous tissue which was situated, in the unoccupied uterus, two or three lines external to the proper uterine orifice. After conception, these parts become infiltrated with serum; or sometimes the venous loops ramifying within them, are distended with blood, or become varicose; and the whole is projected more or less beyond the level of the parts as they existed before impregnation, thus completely obscuring the real *os tincæ*. On examining with the finger, this soft external boundary is very liable to escape notice; the more resisting part of the cervix being sought for, and found higher up in form of a firm ring, dilated certainly, in a very characteristic manner; but not conveying the same idea which the more relaxed external orifice gives when viewed through the speculum. The whole length of the cervix is expanded, from an early period of pregnancy, into a rounded tube, the size of an ordinary writing quill, and in this state it remains, but very slightly altered, until within a month or two of the completion of the term, when it merges itself, by little and little, in the general uterine cavity.

"A circumstance is also noticeable in reference to this subject, of which the touch can take no cognizance, and which, even when the speculum is used, may possibly lead the inquirer into error in cases where the state of the *os* alone is referred to as an evidence of pregnancy. The gelatinous plug which occupies and appears to distend the cervix from an early period after conception, does not remain in that cavity unrenewed: it is always in process of being replaced by a new secretion which is constantly going on; the old deposition being at the same time pushed downwards and dissolved, as it descends, in the vaginal mucus; but in so small a quantity as to escape the notice of the patient under ordinary circumstances. Sometimes, however, the action of the parts is suddenly increased, and the character of the secretion changed. The cervical plug is thus not unfrequently thrown off in a mass, leaving the cavity which contained it unoccupied, and in some measure collapsed, and the external labia fall together. This state of matters, however, is only of temporary duration, as a new supply of mucus is soon furnished, and the cervix becomes distended as before: but if an examination should be instituted at such a juncture, the practitioner, not being aware of the contingency, may be easily led to an erroneous conclusion." P. 207.

Some of Mr. Whitehead's remarks on the Auscultatory evidence of Pregnancy are liable to exception. Few will agree with him in regarding the sound, to which the term *placental souffle* has been improperly applied, as

"an unerring sign." But we shall not say more upon this point, but rather direct our readers' attention to some valuable illustrative remarks which he has made on the subject of *suppression of the menses*, as one of the signs to which undue importance is apt to be attached. He adduces several cases to shew how fallacious this may prove, if accompanied with other indications of the pregnant state. We shall briefly epitomise them.

1. A woman, 29 years of age, had borne six living children at the full term of gestation. In each case, about three weeks after conception, the menses (or, at least, a sanguineous discharge) appeared in unusual abundance, sometimes being mixed with small coagula, and continuing to flow for six or seven days.

2. A young lady menstruated at four successive periods, viz. until the period of quickening, during her first and only pregnancy. She was delivered of a full grown child.

3. A woman, who had had 14 previous pregnancies, "menstruated, to my knowledge, five times during her last pregnancy, which terminated favourably, and at the full period."

4. A woman had a discharge of blood, every way similar to the menses both as to quantity, appearance, and the period of its duration, in the third month of her last (the fourth) pregnancy, which terminated successfully. The discharge returned thrice afterwards, at the end of the fourth, fifth, and sixth months. There was also a leucorrhœal discharge.

5. A woman, who had been long affected with a leucorrhœal discharge, white and glairy before marriage, and yellow and puriform after it, became pregnant with the first child about three years after being married. Menstruation continued in its usual form during the whole period of pregnancy and lactation, and she was unaware of her condition until within a few weeks of delivery, believing herself to be dropsical.

6. In this case, during the fourth pregnancy, "the menses appeared at the accustomed periods, continuing the usual length of time, and, as far as could be ascertained on a superficial examination, bearing the ordinary characters. She was believed to be suffering from internal disease, for the relief of which, after undergoing a course of treatment, change of air was recommended. In due time, at the return of a catamenial period, she was delivered of a fine healthy child at the full term. This patient has, for several years, been troubled with a leucorrhœal discharge, accompanied with bearing down and irritable bladder."

7. A woman, 40 years of age and mother of eight children, was admitted into the Manchester Lying-in Hospital, in the eighth month of her ninth pregnancy. She was menstruating at the time of her admission, and said that she had done so regularly every month since the commencement of the process. She was delivered, a month afterwards, of a full-grown healthy child, the menstrual discharge having appeared two days previously. Menstruation was also regularly repeated during the whole period of lactation; and the same train of phenomena were stated to have occurred in her last, but not in any of her previous pregnancies. She had for three or four years been troubled with leucorrhœa, lumbar and hypogastric pains, bearing-down, and occasional micturition.

Respecting the nature and cause of the discharges in these cases, the reader will, in some measure, have been prepared by what was stated, several pages back, on the subject of *spurious menstruation.* The extract, which we now give, will serve to throw additional light upon the views then propounded :

"A considerable number of cases, similar to the preceding, have occurred to myself, and have been submitted, whenever practicable, to specular examination." P. 222.

"On examination with the speculum, inflammation or ulceration of one or both labia, or of the cervix uteri, complicated, in some instances, with warty excrescences growing from the cervix, or from some part of the vaginal membrane, vaginitis, &c., was met with in every case, without an exception. Fifteen cases were submitted to this kind of examination at the time the blood was flowing. In not one of these did any fluid whatever escape from the interior of the uterus; the orifice being completely occupied at the time by a plug of transparent mucus. On removing the accumulated secretion by means of a piece of lint, the parts were immediately afterwards covered by a coating of blood, which was distinctly seen issuing from innumerable pores on every part of the diseased surfaces, and soon being in sufficient quantity to trickle down into the speculum. This blood was widely different, in its sensible properties, from that collected in the tube during its introduction, or at the os externum ; being more florid, more strongly alkalescent, and soon subsiding into a dryish clot, which could be separated from the interior of the instrument in form of a small cake of crassamentum. This was never the case with the former, which remained fluid or soft, for a considerable time.

"The evidence now produced appears sufficient to establish, as a general rule, to which I am not as yet acquainted with an exception, that the blood discharged in cases of alleged menstruation during pregnancy, is furnished, not by the lining membrane of the uterus, nor by any healthy secreting surface—except sometimes perhaps the inferior part of the inner cervix ; but by the lower extremity of the uterus external to its cavity, or by the contiguous vaginal reflection, being in a state of suppurative inflammation. The fact is always demonstrable by the aid of the speculum. And where specular investigation is found impracticable, there is still no difficulty in forming a diagnosis, so long as the linen of the patient can be submitted to ocular inspection." P. 223.

In a subsequent passage he maintains—

"1. That *menstruation during pregnancy* is, for the most part, perhaps always, associated with an abnormal condition, generally with ulcerative disease, of the uterus ; requiring, at all times, active remedial interference.

"2. That hæmorrhage during pregnancy is not necessarily associated with an altered relation of the parts within the uterus, and, by timely care, need not interfere with the integrity of the ovum.

"3. That menstruation, during the early periods of lactation, is not always normal menstruation, but that it is generally associated with morbid conditions which are amply adequate to the satisfactory explanation of the phenomenon ; that secondary hæmorrhage is, in the majority of instances, not owing to imperfect contraction, or atony of the uterine fibres ; and that the discharge very probably proceeds, under these circumstances, not from the inner surface of the uterus, but from diseased surfaces situated upon parts external to the cavity of the organ." P. 332.

It remains to be seen whether the observations of other obstetrical physicians will warrant the accuracy of these somewhat novel positions.

It is scarcely necessary to say that pregnancy has been repeatedly known to occur before the catamenia had ever made their appearance. Mr. W. relates a good many instances in point. One will suffice ; it is instructive.

"A young woman, seventeen years of age, a dressmaker, was brought to me by her mother, in June, 1844. She had been, for several months previously, in a weak state of health, the principal symptoms being languor, nausea, loss of appetite, and swelling of the belly. She was thought to be suffering from retention, never having menstruated. From some hints that escaped in her relation of a previous course of treatment, which had been administered by a female practitioner, and with which circumstance the mother had been hitherto unacquainted, I expressed my suspicions of the existence of pregnancy; this was strenuously denied, however, by the patient. The breasts were considerably developed, although flaccid; the nipples and areolæ dark and well defined. The abdominal tumour was hard, circumscribed, without fluctuation, and situated low in the cavity. The umbilicus was prominent. No sound was elicited by the stethoscope. An active saline aperient was prescribed. The following morning labour pains came on, and in due time, she was delivered of a still-born fœtus, about seven months grown." P. 225.

### Chap. VI.—*Statistics of Abortion.*

The following table gives the respective periods of abortion in 602 cases, which have occurred in our author's experience. It may be observed that each figure in the first column embraces a period of four weeks, extending from a fortnight before to the same length of time after the month indicated. And, as abortions happening earlier than the seventh week of uterine life are often so nearly simulated by certain menorrhagic discharges, events said to have taken place at this early period—except when an ovum had been clearly made out—have not been included in the report.

| Period of pregnancy at which abortion occurred. | Number of births at each period. | Number still-born. | Number living at birth. | Number living at the end of a month after birth. |
|---|---|---|---|---|
| 2 months | 35 | — | — | — |
| 3 months | 275 | — | — | — |
| 4 months | 147 | — | — | — |
| 5 months | 30 | — | — | — |
| 6 months | 32 | 24 | 8 | 0 |
| 7 months | 55 | 38 | 17 | 3 |
| 8 months | 28 | 23 | 5 | 1 |
| Total | 602 | 85 | 30 | 4 |

It is astonishing how readily abortion is induced in some women, and how strongly it is resisted in others. As an instance of the latter case, our author tells us of "a poor woman who was admitted in the eighth month of pregnancy into the lying-in hospital, having been recently discharged from the Royal Infirmary, where she had been nine weeks an inmate for fracture of the skull, caused by a blow from a hatchet, which she had received in a quarrel. She was delivered of a healthy child, at the full term of gestation."

With respect to the *causes of abortion*, the experience of our author is in strict accordance with the observations of Dr. H. Bennet and other recent writers as to the co-existence of uterine disease in a very great number of cases. This occasionally consists in simple Congestion or vascular plethora of the organ. The symptoms indicative of this state are "immoderate and painful distension of the abdomen, generally attributed by the patients to accumulation of wind in the bowels; a pulsatile movement extending over the whole cavity, the beats being synchronous with the heart's action; sense of weight and bearing-down; intermittent pains of the loins, like those of labour; and, occasionally, escape of blood from the vagina. There is also distension of the pudic, spermatic, hœmorrhoidal, and all the pelvic veins, and sometimes of those of the lower extremities. On examination, the vagina is found hot and turgid, and the cervix uteri tumid and varicose."

But a very much more frequent cause of abortion is disease of the cervix uteri. This is apparent from the following table of the results of our author's observations in 378 cases.

| Accidental agencies. | Placenta prœvia. | Constipation of the bowels. | Retroversion of the uterus. | Incurable disease. | Vascular congestion. | Disease of the lower part of the uterus. | Obscure causes. |
|---|---|---|---|---|---|---|---|
| 44 | 8 | 3 | 3 | 1 | 15 | 275 | 29 |

He subsequently remarks, that "of every hundred cases of abortion, not more than thirty could be attributed by the patients themselves to accidental, or any other appreciable agency: the rest being vaguely referred to a weak state of health, especially to the condition denominated an 'inward weakness,' to previous abortion, difficult labour, protracted recovery, and, in some instances, to diseases of a specific nature. The two hundred and seventy-five individuals ranged under this head in Table X., were, with a very few exceptions, examined with the speculum, either before, or within three or four weeks after the event took place; and in every case thus submitted to examination, disease of the lower, or of the internal part of the uterus, and in a few instances, of the vagina, was found to exist. Some of those, who had the disease in a severe form and of long standing, underwent the necessary course of treatment at the time, and recovered: but a great number disappeared after being two or three times prescribed for; unwilling, apparently, to believe that further attention was necessary."

The forms of uterine disease said to predispose to or to induce abortion, are inflammation and superficial erosion of its mouth and cervix; varicose ulceration of one or of both labia of the *os tincæ;* œdema of the cellular structure of the cervix; fissured ulceration of one or both commissures, of the anterior or posterior labium, or implicating all these parts at the same time, together with inflammatory hypertrophy of the adjacent structures; induration of the cervix, with or without abrasion of surface; endo-uteritis, or inflammation of the lining membrane of the uterus; follicular ulceration; gonorrhœal inflammation of the os and cervix, often extending into the cavity of the uterus; syphilitic disease, both in its primary, secondary, and tertiary stages; and, lastly, prolapsus of the uterus.

The symptoms denoting a diseased condition of the lower part of the uterus are the presence of a leucorrhœal discharge, whether the matter discharged be simply mucous, or have an admixture of pus or blood; a deep-seated uneasiness in the hypogastrium; a fixed pain in one or in both inguinal regions; aching of the loins; a sense of distressing "bearing-down;" rigors, lassitude and remittent feverishness. There is often also some uneasiness or other inconvenience in passing water, hæmorrhoids, an acute smarting, or stabbing pain about the coccyx, accompanied with a feeling akin to tenesmus, nausea, loss of appetite, irregularity of bowels, cramps, palpitations, hysteric fits, convulsions, &c. Mr. Whitehead describes with great minuteness the various kinds of leucorrhœa, and points out the different conditions of the uterus and vagina which they serve to indicate. Whenever there is any admixture of puriform matter with the discharge, there is strong reason to suspect some diseased condition of the lower part of the uterus.

So frequent is leucorrhœa in one form or another during pregnancy that it was found by Mr. W. to be present in 1116 out of 2000 women; and in 936 of the former number, or 83 per cent., the discharge bore undoubted evidence of the presence of pus or of sanies, and occasionally of blood: of these, 544, or 58 per cent., had previously miscarried. The following data will be read with interest:

"Of forty-five women pregnant for the first time, all suffering under leucorrhœal affections, twenty-eight had the discharge of a decidedly purulent character. I examined the uterus in twenty-five of these, and all, with but one exception, had ulcerative disease of the lower part of the uterus; in the exceptional case, the vaginal membrane was studded with warty excrescences which were known to have had a syphilitic origin. I examined several of those also, who declared the discharge to be colourless, and found suppurative disease to be equally prevalent in them. Of more than two thousand individuals labouring under leucorrhœal affections, in whom I have examined the uterus with the speculum, I have, with comparatively few exceptions, found the existence of structural lesion sufficient in degree to account for all the symptomatic phenomena. In the great majority of the cases, the cervix and labia uteri were the seat of disease; in some there was excoriation, erysipelas, high vascular congestion, or a warty state of the vagina; in others, the labia, free from ulceration, were thickened, with their inner margins callous and flabby; or else they were tense, and presenting a vivid redness around the orifice of the uterus; both which appearances indicate the existence of disease within the organ." P. 287.

We may remark, *en passant*, that the puriform leucorrhœal discharge may give rise to blenorrhagia in the husband. The infant too, born under such circumstances, is apt to be affected with purulent ophthalmia, and other affections of a similar nature.

We strongly suspect that a good deal of the description which our author gives of *Endo-uteritis* (the name tells the nature and seat of the disease) is drawn from fancy:

"The form of disease now proposed for consideration is one of a strictly local character, of every-day occurrence, and very amenable to treatment. It nevertheless acts as a common cause of abortion during the early months of pregnancy; and it constitutes, in the majority of instances, the pathology of that species of disordered or difficult menstruation known as dysmenorrhœa. It does not occur as a necessary consequence, however, that dysmenorrhœa, as commonly witnessed in early life, indicates the existence of a condition likely to create an inaptitude for child-bearing afterwards; on the contrary, the symptoms in the virgin are often of a purely nervous, or what is understood in common parlance, of an hysterical character, unaccompanied with inflammatory action, and they frequently undergo complete cure by marriage: an instance of which is given in Case XVI., illustrative of the article on this subject.

"Endo-uteritis is a term employed to signify inflammation of the lining membrane of the uterus. The affection sometimes implicates the cavity of the cervix, or that of the lower part of the organ only; at other times the whole lining membrane is involved, and it not unfrequently extends within the Fallopian canals to their outer extremities. The inflammation is generally of a chronic, although of a very irritable character; and under certain states of excitement, as febrile irritation resulting from the application of cold, or of accidental violence; inordinate venereal indulgence; the action of the gonorrhœal or syphilitic virus, &c.; the deeper textures of the uterus and neighbouring organs may be seriously implicated." P. 352.

The symptoms of this morbid condition of the uterus are alleged to be "distension of the hypogastrium, accompanied with a constant, deep-seated aching behind the pubis; irritable bladder; pain of the loins, of the inguinal regions, and of one or both sides of the abdomen on a level with the umbilicus; languor; irritative fever; and vaginal discharge. The whole uterus is often found in a state of inflammatory hypertrophy, and, unlike the affections previously noticed, is extremely painful upon pressure, especially at the *back part of its body*, where it impinges upon the rectum. The cervix is hard and less sensitive, but slight succussion made upon this part develops a painful sensation about the inguinal or umbilical regions, or across the loins. Examined with the speculum, the labia present a tense, glistening appearance, and a ring of vivid redness surrounds the orifice; this, in some cases, is seen to extend upon the surface of the *posterior* lip. Sometimes one or both labia are excoriated, eroded, or fissured."

Accumulation of air within the uterus (*physometra*) is said to frequently accompany inflammation of this organ. "It is commonly discharged in the form of bubbles, which may often be seen to form and burst in rapid succession during specular inquiry. There is reason to believe that it sometimes collects in considerable quantity, causing great suffering by distension of the uterine walls, and being expelled by sudden contraction of the organ, accompanied with severe forcing pains like those of labour. Generally speaking, it emits an offensive odour, owing in most instances, probably, to decomposition of the small coagula liable to be retained after menstruation, or which may also be thrown out at other times. It may possibly be, on some occasions, the product of secretion."

Mr. Whitehead asserts that abortion is, in many cases, owing to inflammation of the lining membrane of the uterus, resulting in an imperfectly organised condition of a portion of the decidual membrane.

The following passage, professing to give a description of *Gonorrhœal Inflammation of the Uterus*, will be found to contain statements which will require confirmation, before they command general assent.

"Gonorrhœa in the female is much more frequently an affection of the uterus than of the vagina. This, although totally at variance with the opinions hitherto entertained, is nevertheless what might reasonably have been anticipated. In the first place, the gonorrhœal virus, from physiological causes, is liable to be carried immediately to the highest part of the canal, and forcibly projected upon the lower extremity of the uterus, which organ also, at this juncture, is in a state eminently calculated speedily to absorb it; in the second place, the normal secretion of the vagina possesses properties which are capable, to a certain extent, of destroying, or of materially modifying the virulency of the morbid product, and of thus protecting the vaginal surface from its immediate influence. The urethral orifice, however, seems to be provided with this means of protection in a much less perfect degree, and is, therefore, more highly susceptible of the action of specific inoculation. In nine unimpregnated women afflicted with gonorrhœa, seven had inflammation, with abrasion of the labia uteri, and in the remaining two, the inflammatory action was confined to the vaginal surface; the parts beneath the arch of the pubis being most severely affected: in one of these the urethral orifice was also involved.

"The first change operated upon the uterus after gonorrhœal inoculation, consists in superficial inflammation of one or both labia at their most depending part, or at the boundary of the os and commencement of the internal cervix. The inflammation seems to affect principally the small mucous follicles with which the surface is closely studded—(probably not the Nabothean bodies already noticed). A small red patch is first perceived; sometimes there are two or three isolated spots which extend and soon run together, forming one patch, of variable size in different cases and in different stages of the complaint, and generally of irregular shape. On removing the thick secretion with which this is covered, the surface appears to consist of minute granules, equally dispersed over every part of it; the abrasion is bounded by a margin not very distinctly defined, running imperceptibly into the erysipelatous redness which surrounds the sore; this extends to some distance upon the cervix, the whole of which is more or less tumid, but not painful to the touch." P. 362.

He subsequently adds:—

"I have seen no small number of cases of this disease, and have almost invariably found the lower part of the uterus inflamed or ulcerated; where the urethra or the adjacent structures have been involved, the affection of these parts has been proved to be of secondary character, resulting from a transfer of the morbid product from the seat of the primary inoculation—the uterus." P. 365.

Gonorrhœal inflammation is said to be extremely liable to extend within the cavity of the womb, and give rise to chronic Endo-uteritis; a distressing affection, which may exist for years, "rendering the whole of each succeeding pregnancy a period of suffering and misery, in many instances occasioning abortion at different stages of the process; sometimes it effectually prevents the accomplishment of impregnation, and it is not an uncommon cause of dysmenorrhœa. The symptoms are always aggravated during pregnancy; the inflammation involving the deeper textures of the cervix, and resulting in interstitial deposit, and consequent induration."

Our author seems to be of opinion that a very large proportion of the cases of ulceration of the uterine mouth and neck are of gonorrhœal origin. We need scarcely say that, in his opinion, the application of the nitrate of silver and other astringents must be made directly to the os and cervix uteri in the treatment of gonorrhœal inflammation. He says that the patient herself may be taught how to apply any fluid remedy, with the aid of a speculum. Injections he declares to be of little use.

Passing over the subject of Syphilitic disease of the uterine orifice, we shall briefly notice one or two points in our author's description of *Prolapsus uteri.* He maintains that this disease of the organ, like abortion, is more frequently owing to inflammation and ulceration of the lower part of the organ than to any other cause. With respect to the treatment of this very distressing affection, Mr. Whitehead expresses his strong objection to the use of pessaries in the generality of cases; nay, he goes so far as to assert that "they invariably aggravate the disease for the relief of which they are employed." After pointing out the necessity of attending to the general or constitutional health of the patient, he remarks:—

"The local treatment should consist in applications of nitrate of silver, or other suitable remedies to the diseased surface, and in the insertion of medicated tents by the aid of the *Prolapsus tube.* This latter procedure may be practised immediately after the nitrate has been applied, although the remedy with which the tent is charged be of a very different nature from that of the caustic.

"The manner of using the *Prolapsus tube*—which will be found of equal service, in the management of prolapsed displacement of the uterus, as in most other forms of uterine disease, and enables the patient safely and efficiently to apply the remedies herself, without the interference of the practitioner—is extremely simple. The charged tent, to which a length of thread has been previously attached in the manner before directed, must be placed in the tube, the upper orifice of which is to be applied against the protruded portion of the uterus, in such a manner as to receive the os uteri within it. The instrument, previously smeared with some unctuous material, and having its curved arm placed anteriorly, in a direction towards the abdomen, is now to be forced gently and steadily backwards, until the whole, or greater portion of it, has passed within the canal, or until a moderate degree of resistance is felt to oppose its further ingress." P. 394.

"The uterus being thus restored to its natural position, the tent or pledget must be pressed upwards against the cervix, and held in that situation by means of a skewer or other suitable instrument, the tube at the same time being gently withdrawn. The recumbent posture should be strictly maintained for several days, and very little exercise taken for some weeks afterwards. The cases recorded in the accompanying table were those of poor women, obliged, during treatment, to pursue their domestic duties, which constantly and seriously interfered with the favourable effects of the remedies; hence the reason, probably, why most of them were so long under treatment. The application should be renewed daily, or twice a-day if convenient." P. 395.

The lotions, used for moistening the lint-tent, are strong solutions of nitrate of silver, sulphate of zinc, sulphate of copper, matico, opium, and tannin. The metallic proportions should not be employed oftener than every third or fourth day, the vegetable applications being used intermediately. An emollient injection should be made use of after the removal of each tent.

We shall now briefly advert to the contents of Mr. Whitehead's last chapter, that which professes to treat of Sterility. His remarks upon the fatal influence of abnormal states of the vaginal mucus on the vitality of the spermatic animalcules need not detain us; most of them are mere conjectures; not his, by-the-bye, but those of M. Donné whom he quotes. As to the morbid conditions of the uterus, which he enumerates among the causes of Sterility, endo-uteritis—*endo-metritis* would be a better word —and ulcerative inflammation of the cervix are declared to be the most frequent. It will be our best plan briefly to analyse a few of the cases adduced by Mr. W. in illustration of his views :—

1. A woman, aged 41, was admitted into the Manchester Lying-in Hospital, eight months advanced in her second pregnancy. She had been married when 18 years of age, and borne her first child a year and a half subsequently. After this period, she was seldom free from leucorrhœa, which was often of an acrimonious character, during the menstrual intervals. The catamenia were tolerably regular, but always attended with great suffering. Her husband survived sixteen years; and, a twelvemonth after his death, she married a second time, her health having somewhat improved in the interim. "Two years and three quarters after marriage she experienced an attack of fever, for which she was an in-patient of the Manchester Fever Hospital, after which she enjoyed excellent health: 'the fever having set her up.' All symptoms of uterine disturbance disappeared on the accession of the febrile affection, and did not afterwards return. Pregnancy took place eight or ten weeks after convalescence, and she was delivered in due time of a living child at the full term of utero-gestation. The uterus, which was examined previous to her dismissal from the Lying-in Hospital, exhibited traces of former disease, but was perfectly healthy."

We may pass over the next case, as there was unfortunately no "cure" effected. The one that follows is headed thus: *Sterility fifteen years; secondary syphilitic affection of the uterus of sixteen years' duration; sanious leucorrhœa; painful menstruation; pregnancy; cure.* By-the-bye, is not the cart put before the horse here?—was not the cure before the pregnancy? The circumstances of the case are these. A young woman was infected with syphilis by her husband soon after marriage; she was never pregnant by him. He died twelve years afterwards; and then, after a twelvemonth's widowhood, "being then in a better state of health," she married a second time. Her general health became much improved, and, as might be expected, the leucorrhœal discharge, with which she had been affected for many years, was much diminished. Two years subsequently, she became pregnant for the first time. As she was threatened with abortion soon after quickening, she consulted Mr. Whitehead. He found, on specular examination, the following appearances :—

"The cervix uteri was unusually large and firm; the boundary of the orifice was covered with granulations which appeared to extend to the interior of the organ; the outer boundary of this ulcer was marked by a raised margin, beyond which the surface of the cervix was of a dark-red colour and mottled; a fissure occupied the right commissure, from which and from the adjoining granulations blood was exuding. On communicating my suspicions respecting the syphilitic nature of the complaint under which she was labouring, her history as above given was related in a straight-forward manner; but she was unwilling to believe that any trace of the venereal affection remained in her constitution, so that no treatment was adopted having reference to that particular purpose. Her delivery took place on the 11th of July, 1846, at the full period of utero-gestation." P. 411.

The child subsequently exhibited indubitable traces of venereal taint.

The following case is certainly more satisfactory than either of the preceding :—

A lady, married at the age of 21, became affected a few weeks after marriage with purulent leucorrhœa, which was suspected to be of gonorrhœal origin and treated accordingly. After the active symptoms were subdued, the discharge continued, more or less aggravated from time to time by exposure to cold, or after severe exercise; it was, also, always augmented a few days previous to each return of the catamenia. Menstruation was attended with greater suffering than formerly; the secretion was more abundant, continued several days longer than it was wont to do, being sometimes clotted, or grumous and offensive, and occasionally, towards the end of the period, mixed with membranous substances.

This unpleasant state of things had continued, with varying degrees of severity, for six years: she had never been pregnant. The general health was much impaired. The local malady is thus described:—"The whole uterus was found in a state of inflammatory hypertrophy, painful under pressure; the cervix was slightly indurated; and an angry-looking granulating ulcer of irregular shape occupied the lower and inner parts of both labia, and appeared to extend within the orifice of the uterus, whence flowed a thin ichorous secretion."

Although the ulcer is here described as *angry-looking*, we subsequently read that the speculum was used for the first time about a twelvemonth afterwards. How is this discrepancy to be accounted for? Under the use of alterative and tonic medicine, with the addition of astringent injections, the leucorrhœal discharge became considerably diminished, and the general health was much improved. Six months later, she miscarried in the third month of pregnancy: this was in March. In the following November she again miscarried, "at the end of the 5th month." The rest of the report we shall give in our author's own words:—

"She now expressed an anxiety about her case, and begged that the necessary means might be adopted for its efficient cure. The leucorrhœal affection was said to have become considerably aggravated previous to miscarriage, and appeared to be in a similar state a fortnight afterwards when the treatment was commenced. On the introduction of the speculum, which was now used for the first time, the cervix appeared unusually large, hard, exhibiting several patches of excoriation; two deep ulcerated fissures occupied, one the left commissure, the other the middle of the posterior labium; the orifice was surrounded by a bright red, inflamed surface, and the whole was covered with a sanio-purulent secretion, which became considerably mixed with blood during examination. The nitrate of silver was freely applied to all the diseased surfaces, as well as within the cervix, and was afterwards several times repeated at intervals of six or seven days. She also resumed the use of iodide of iron combined with extract of cinchona. At the end of eleven weeks, the cure was complete: every part of the uterus was perfectly healed and of normal dimensions and consistence; the mind was cheerful and happy, the health vigorous; and, for the first time since marriage, she was free from leucorrhœa.

"She was delivered of a living child at the full period of utero-gestation, on the 25th December, 1846." P. 418.

In the case that is next reported there was obstinate leucorrhœa of many years standing, and there was presumed to be "ulceration of the cervix uteri and probably endo-uteritis;" the speculum was not used. A strong solution of the nitrate of silver—ʒj. to ℥vj. of water—was ordered to be used as an injection, directions being given that, in using it, the syringe should be introduced as high up as possible. After being employed five times, severe inflammation of the vagina came on; but this, in the course of a few days, subsided, and the patient improved rapidly. She afterwards spent a few weeks in the country, and returned in better health than she had experienced since marriage. The so-called mistake had completely cured the leucorrhœal affection, and consequently the uterine disease also: she shortly became pregnant, and was delivered of a living child at the full term of utero-gestation, eleven months after the injection had been used.

In the case that follows this one, the symptoms of chronic endo-uteritis were mistaken, our author says, by several practitioners for those of "spinal irritation;" in consequence of there being, along with a fixed uneasiness in the left groin and an almost constant leucorrhœal discharge, "a violent pain, extending from the third dorsal vertebra to the sacrum." Upwards of four years had passed since the lady had miscarried about six months after marriage; she had never been pregnant again. "The lower part of the uterus was in a state of inflammatory hypertrophy; the labia were thickened and projecting, and presented the ring of vivid redness around the orifice indicative of internal inflammation. To these parts the solid nitrate of silver was freely applied; leeches were applied alternately to the sacral and hypogastric regions, and remedies of an alterative character were administered. At the end of three months, her condition being remarkably improved, although the leucorrhœal discharge was still present, she became pregnant." It seems, however, that, as the pregnancy advanced, all her former distress returned. "Soon after quickening, attacks of intermittent pains similar to those of labour, came on, the discharge was mixed with blood, and my attendance was solicited for the purpose of facilitating delivery, which was considered as being near at hand. The cervix uteri was large, inflamed, and excoriated, and a granulating surface occupied the boundaries of the orifice. To all these parts the nitrate of silver was again freely applied; two grains of opium combined with an equal quantity of *hydr. subm.* were given, and the strictest quietude enjoined. On the following morning she was free from pain: she had scarcely slept at all, but had been perfectly tranquil the whole night. Anodyne remedies and applications of caustic, with due attention to the alvine functions, were found occasionally necessary during the remainder of the process, which terminated in the birth of a living child of full growth on the 5th of April, 1846."

Subsequently, there was a renewal of the symptoms that had so long affected the health. Believing that it was the internal surface of the uterus that was the primary and chief source of the mischief, Mr. Whitehead ordered a weak solution of the nitrate of silver to be forcibly injected into its cavity. It caused no pain or inconvenience, and its use was followed by the most gratifying results. The operation was repeated ten days afterwards. "The first injection was introduced on the 24th September, since which, now more than eight months, she has not had the least symptom of her former or of any other ailment, nor has she since taken a single dose of medicine. She has the appearance of one in robust health."

These cases will doubtless suggest some useful hints to the reflecting practitioner, and the successful treatment in two or three of them is highly creditable to the skill of our author. He cannot, indeed, claim any originality as to the discovery of the connection that so often exists between ulcerative inflammation of the lower segment of the uterus and Sterility; for on this point, as indeed on most others treated of in the volume before us, he has been quite anticipated by Dr. Henry Bennett, to whom unquestionably belongs the almost exclusive merit of having, within the last few years, drawn the attention of the profession—in this country at least—to the important part which this morbid state plays in the production of some of the most troublesome complaints to which women are subject, both in the pregnant and the unimpregnated condition. Mr. Whitehead seems, rather strangely, to be but very imperfectly acquainted with the results of his predecessor's researches. The following propositions, with which Dr. Bennett concluded a series of very interesting and valuable papers which appeared in the *Lancet* during the course of last year, may be appropriately introduced here. Their general accuracy has been strikingly confirmed by the subsequent enquiries of our author as well as of other observers.

"1. Inflammatory ulceration of the uterine neck is not an uncommon disease in the gravid uterus, although hitherto entirely overlooked by uterine pathologists and by accoucheurs.

"2. When this disease exists in the pregnant state, its symptoms are the same as in the non-pregnant condition, but obscured, and more or less modified, by the pregnancy.

"3. It is a frequent cause of disordered health during pregnancy, or of 'laborious pregnancy.' It is also one of the most frequent causes of Abortion, both in the early and in the later months of pregnancy. It may occasion abortion, either directly, by reflex action, or indirectly, by giving rise to disease of the ovum or placenta, or to uterine hæmorrhage.

"4. The instrumental examination of females labouring under inflammatory ulceration of the cervix during pregnancy is unattended with any risk, either to the mother or to the fœtus; and it is absolutely necessary, in order not only fully to recognise the disease, but also to treat it.

"5. The treatment of these forms of uterine inflammation must be governed by nearly the same rules in the pregnant state as in the non-pregnant state. A properly conducted treatment is nearly always successful in preserving the life of the child and the integrity of the pregnancy, as well as in curing the inflammatory and ulcerative disease. It is also the only means we possess of warding off the imminent danger of abortion to which the patient is exposed.

"6. This form of uterine inflammation being, generally speaking, the cause of those repeated and successive miscarriages which prevent females giving birth to a living child, it is only by curing it that we can hope to make them bear the product of conception to its full period.

"7. The serious inflammatory and hæmorrhagic symptoms which sometimes follow abortions are generally occasioned by unrecognised inflammatory ulceration of the uterine neck. On investigation, it often becomes evident that this disease existed previous to the abortion, and occasioned it. The same remark may apply to some cases in which the above-mentioned symptoms precede and follow labour at the full time, as ulcerative inflammation of the cervix in the pregnant state is by no means *necessarily* followed by abortion.

"8. Although inflammatory ulceration of the cervix seems generally to be a cause of Sterility, yet, as will appear from the above essay, there are frequent exceptions to the rule. In some females, the tendency to become impregnated is so great that no amount of uterine disease appears to prevent conception taking place."

In taking leave of our author we must again enter our *caveat* against all uncalled-for or unadvised use of the speculum in the complaints of females. Though most valuable in many cases, and absolutely necessary in others, it need scarcely be said that the employment of this means of exploration *may* become the source of no trifling mischief, in more ways than one. We cannot but think that Mr. Whitehead has set a very questionable example to his younger brethren in the profession, by his frequent adoption of it in that condition of the female system, when the instinctive feelings of Nature shrink from exposure; nor can we think very highly of an author's feelings of decorum, who could deem it at all necessary or proper to proclaim to the public his belief "that the danger of copulation during the period of menstruation to be in a great measure assumed and fanciful, or, at all events, to be far less imminent than has been represented."

If Mr. Whitehead's volume reaches a second edition—which we think not unlikely, considering the value of a great part of its contents—it must undergo a very thorough revision, in the way of retrenchment, compression, and collation with what has been done by other writers in the same department of pathological enquiry. We would particularly direct his attention to a very valuable paper from the pen of Dr. Evory Kennedy in our excellent cotemporary, the Dublin Medical Journal, for last May.

### ERRATUM.

Dr. E. Kennedy's paper, referred to in page 405, appeared in the number of the Dublin Medical Journal for February, not May, last.

